# A comparative analysis of L1 retrotransposition activities in human genomes suggests an ongoing increase in L1 number despite an evolutionary trend towards lower activity

**DOI:** 10.1186/s13100-021-00255-x

**Published:** 2021-11-15

**Authors:** Sawsan Sami Wehbi, Heinrich zu Dohna

**Affiliations:** Department of Biology, American University of Beirut, Beirut, Lebanon

## Abstract

**Background:**

LINE-1 (Long Interspersed Nuclear Elements, L1) retrotransposons are the only autonomously active transposable elements in the human genome. The evolution of L1 retrotransposition rates and its implications for L1 dynamics are poorly understood. Retrotransposition rates are commonly measured in cell culture-based assays, but it is unclear how well these measurements provide insight into L1 population dynamics. This study applied comparative methods to estimate parameters for the evolution of retrotransposition rates, and infer L1 dynamics from these estimates.

**Results:**

Our results show that the rates at which new L1s emerge in the human population correlate positively to cell-culture based retrotransposition activities, that there is an evolutionary trend towards lower retrotransposition activity, and that this evolutionary trend is not sufficient to counter-balance the increase in active L1s resulting from continuing retrotransposition.

**Conclusions:**

Together, these findings support a model of the population-level L1 retrotransposition dynamics that is consistent with prior expectations and indicate the remaining gaps in the understanding of L1 dynamics in human genomes.

## Introduction

Transposable elements (TEs) are mobile DNA segments that comprise more than half of the human genome [1]. They are classified as short interspersed elements (SINEs), long interspersed elements (LINEs) or long terminal repeat elements (LTR elements). L1s are a class of LINEs and are the only active autonomous retrotransposons in humans. Their dynamics are poorly understood.

De novo L1 insertions can be disruptive to the host genome. Some specific insertions have been linked to diseases such as hemophilia and thalassemia [2]. Overall high rates of retrotransposition may increase the risk of proliferation and metastasis of epithelial cancer [2] and have been associated with the psychiatric disorder schizophrenia [3]. Despite disruptive effects of L1 insertions, L1s have become an integral part of their hosts’ developmental process. In mice, L1 transcription regulates chromatin accessibility during embryogenesis, which is an integral for proper mouse embryo development [4]. Neuronal mosaicism due to L1 retrotransposition has recently been suggested to play a role in learning and memory [5].

Although L1s have profound effects on their host, the dynamics of L1s in the human population are still poorly understood. Different L1 families appear to have expanded in the human lineage at different times [6], but it is unknown whether the number of L1 insertions is currently growing in the human genome. The L1 dynamics are determined by the balance between the rate at which new insertions are generated due to continued retrotransposition, and the rate at which insertions are removed due to selection [7, 8]. This balance is complicated by the fact that the retrotransposition rate itself can evolve. Full-length L1s in the human genome show a considerable variation in retrotransposition activity [9].

Selection on the retrotransposon level should lead to an increase in the retrotransposition rates since more active L1s generate more insertions, which tend to be more active themselves. Without selection, one would expect that after insertion the retrotransposition activity of an L1 decreases over time because random mutations of L1 sequences are more likely to disrupt the retrotransposition machinery rather than improve it. In addition, host-level selection is likely to favor low retrotransposition activity due to the general disruptive effects of retrotransposition for the host, leading to a scenario of decreasing retrotransposition activity post insertion. While this scenario is plausible, it is also conceivable that host-level selection maintains retrotransposition, due to potentially beneficial effect of retrotransposition for the host. We are not aware of any study that compared these scenarios for the evolution of L1 retrotransposition rates with data from human genomes.

The evolution of the retrotransposition rate can only be understood if the retrotransposition rate can be reliably estimated for individual L1 sequences. Retrotransposition activity of individual L1s is usually measured in cell culture [9–11]. It is unclear how well the cell-culture-based rates approximate the rate of in vivo L1 insertion in the germline. Transduced sequences have been used to estimate parent-offspring relationships among L1 insertions in the human reference genome and thereby infer retrotransposition rates [12]. Cell-culture based retrotransposition activity estimates are weakly correlated with L1 insertion rates inferred from transduced sequences [12]. Retrotransposition rates that are inferred from transduced sequences are confounded by the age and allele frequency of L1s. Other approaches are needed to improve our understanding of the relationship between cell-culture based retrotransposition activity and L1 germline insertion rates.

Comparative methods can be used to estimate the rates at which new L1s emerge in the population. However, it is likely that these population-level rates of L1 emergence do not only depend on intrinsic retrotransposition rates but also on the relative importance of selection and drift, which in turn is influenced by the effective population size. Since the human effective population size underwent some dramatic changes, it is important to account for population-level temporal trends when estimating intrinsic L1 transposition rates through comparative methods.

In this study we use comparative methods to address four interrelated questions about the evolution of retrotransposition activity, namely: (i) do L1s with a higher cell-culture measured retrotransposition rate generate more new L1 insertions detectable in the human population? (ii) is there an evolutionary trend towards a lower intrinsic retrotransposition rate after insertion? (iii) is there a population-level temporal inhomogeneity in the overall retrotransposition rate and (iv) what is the L1 growth rate in human genomes resulting from estimates obtained from addressing questions (i) - (iii)?

## Materials & methods

### Sequence collection and alignment

The nucleotide sequences of 155 full-length L1s in the human genome were obtained from two studies that published L1 sequences and their corresponding retrotransposition activity values [9, 10]. The L1 sequences from Brouha et al. [9] were extracted from an alignment provided in their supporting information. The L1 sequences from Beck et al. [10] were obtained by identifying from their supporting information the sequences flanking L1 insertions, and locating these flanking sequences in the corresponding fosmid sequences. The L1 nucleotide sequences were aligned using MAFFT [13].

### Tree reconstruction

Phylogenetic trees of L1s were reconstructed from L1 sequence alignments using BEAST v.10.4 [14] to fit a general time reversible substitution model with a combination of a gamma distribution for rate variation among sites and a proportion of invariant sites. The prior distribution of tree branch lengths was calculated according to a Yule birth process.

### Binary State Speciation and Extinction (BiSSE) model

A Binary State Speciation and Extinction (BiSSE) model, which combines a speciation-extinction model with the two-state Markov model [15, 16], was fitted to the L1 phylogenetic trees. The L1 retrotransposition activities were coded as a binary character, where L1 alleles with retrotransposition activities below or above 25% of the reference L1_RP_ were classified, respectively, as low or high retrotranspositon L1s. According to the BiSSE model, the two character-states (high and low retrotransposition activity in our case), evolve along the tree based on a Markov process, and the speciation and extinction rates of lineages depend on the character-states, leading to six parameters (Table 1). In this context, the speciation rates correspond to the rate of emergence of new L1 insertions whereas the extinction rates correspond to the rate at which L1 insertions are removed from the population. The R package diversitree [17] was used to calculate the likelihood of the data, given the BiSSE parameter values and a phylogenetic tree. To obtain a likelihood value that accounts for uncertainty in tree estimation, the tree-specific likelihoods were summed over a sample of 150 trees from the posterior distribution of trees generated by BEAST. The parameter values maximizing this likelihood function were estimated using the R function constrOptim. Eight models with different parameter constraints were fitted to the data (Table 2). The fit of these models was compared according to the Akaike information criterion (AIC). The parameter constraint that achieved the best fit among the eight models was chosen to construct two additional models that included either a single change point at 140 generations ago or two change points, at 140 and 4720 generations ago. The later change points correspond to the estimated start of a recent population expansion and the earlier to the end of a human population bottleneck [18]. At each change-point the two speciation rates (λ_0_ and λ_1_) can change by a factor that is the same for both speciation rates (λ_0_ and λ_1_). Hence, these models add one additional parameter per change point. BiSSE measures branch lengths in proportion of nucleotide substitution whereas the population genetic events in the change point model were specified in number of generations ago. To convert between these units, a substitution rate of 2.5*10^− 8^ per nucleotide and generation was used [19].

**Table 1:** Parameters of the speciation-extinction model fitted to L1 phylogenetic tree with binary retrotransposition activity data

Parameter	Description
λ_0_	Speciation rate of low activity L1
λ_1_	Speciation rate of high activity L1
μ_0_	Extinction rate of low activity L1
μ_1_	Extinction rate of high activity L1
*q*_01_	Rate at which low activity L1 change into high activity L1
*q*_10_	Rate at which high activity L1 change into low activity L1

**Table 2:** Results of speciation-extinction models fitted to L1 phylogenetic tree with binary retrotransposition activity data

Constraint	Df	Parameter values	Log likelihood	AIC
Unconstrained	6	λ_0_ = 10, λ_1_ = 412, μ_0_ = 0, μ_1_ = 0, *q*_01_ = 30, *q*_10_ = 359	609.54	− 1207.09
μ_0_ = μ_1_	5	λ_0_ = 10, λ_1_ = 412, μ_0_ = μ_1_ = 0, *q*_01_ = 31, *q*_10_ = 360	609.54	− 1209.09
λ_0_ = λ_1_	5	λ_0_ = λ_1_ = 167, μ_0_ = 0, μ_1_ = 0, *q*_01_ = 30, *q*_10_ = 211	550.67	− 1091.35
*q*_01_ = *q*_10_	5	λ_0_ = 14, λ_1_ = 1510, μ_0_ = 0, μ_1_ = 1399, *q*_01_ = *q*_10_ = 159	587.12	− 1164.23
μ_0_ = μ_1_ = 0	4	λ_0_ = 10, λ_1_ = 412, *q*_01_ = 31, *q*_10_ = 360	609.54	− 1211.09
λ_0_ = 0	5	λ_1_ = 403, μ_0_ = 0, μ_1_ = 0, *q*_01_ = 42, *q*_10_ = 370	606.65	− 1203.3
*q*_01_ = 0	5	λ_0_ = 9, λ_1_ = 361, μ_0_ = 7, μ_1_ = 0, *q*_10_ = 275	607.43	− 1204.86
μ_0_ = μ_1_ = λ_0_ = 0	3	λ_1_ = 406, *q*_01_ = 43, *q*_10_ = 372	606.65	−1207.3
μ_0_ = μ_1_ = *q*_01_ = 0	3	λ_0_ = 9, λ_1_ = 357, *q*_10_ = 270	607.42	− 1208.83

### Asymptotic L1 growth rate

The parameters of the BiSSE model define the following system of differential equations:

$$\frac{d\boldsymbol{x}}{dt}=\left[\begin{array}{cc}{\lambda}_0-{\mu}_0-{q}_{01}& {q}_{10}\\ {}{q}_{01}& {\lambda}_1-{\mu}_1-{q}_{10}\end{array}\right]\boldsymbol{x}$$

where the vector ***x*** denotes the number of low and high activity L1s. The dominant eigenvalue of the matrix in the above equation gives the asymptotic rate of increase of L1 insertions and the associated eigenvector the relative number of high and low activity L1s in the stationary phase.

### Bayesian transition model estimation

The posterior distributions of evolutionary rates between the two retrotransposition activity states along the phylogenetic trees were also estimated using the Bayesian MCMC software package BayesTraits [20]. The posterior distribution of trees generated by BEAST was used as input data for the transition model analysis. The posterior probabilities of different model constraints were estimated via reversible jumps.

## Results

**Fig. 1:**
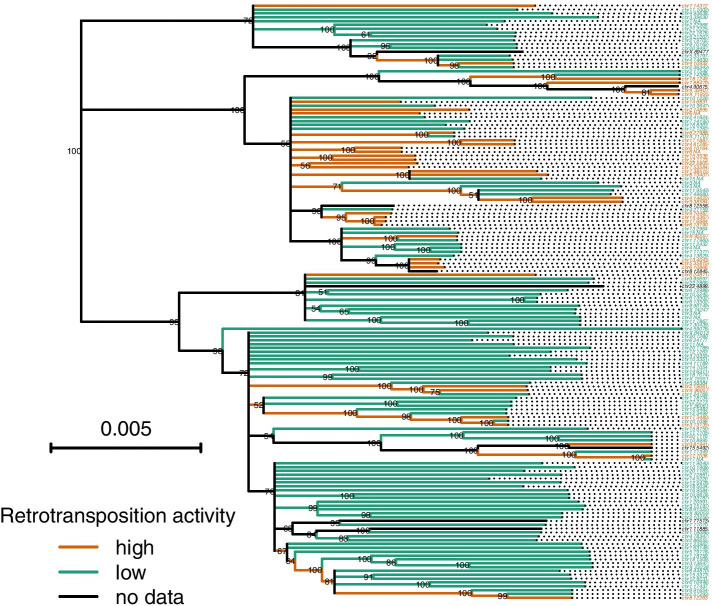
Consensus phylogeny based on 155 published L1 sequences with estimated retrotransposition activity. Tree branches are colored by retrotransposition activity and node labels show posterior probability values. The tree was rooted using the consensus ancestral sequence of L1PA2. The tree was estimated using BEAST. The tip labels show for each L1 the chromosomal coordinates (left side) on the reference genome hg38. NAs indicate L1s whose genomic position could not be determined

**Fig. 2:**
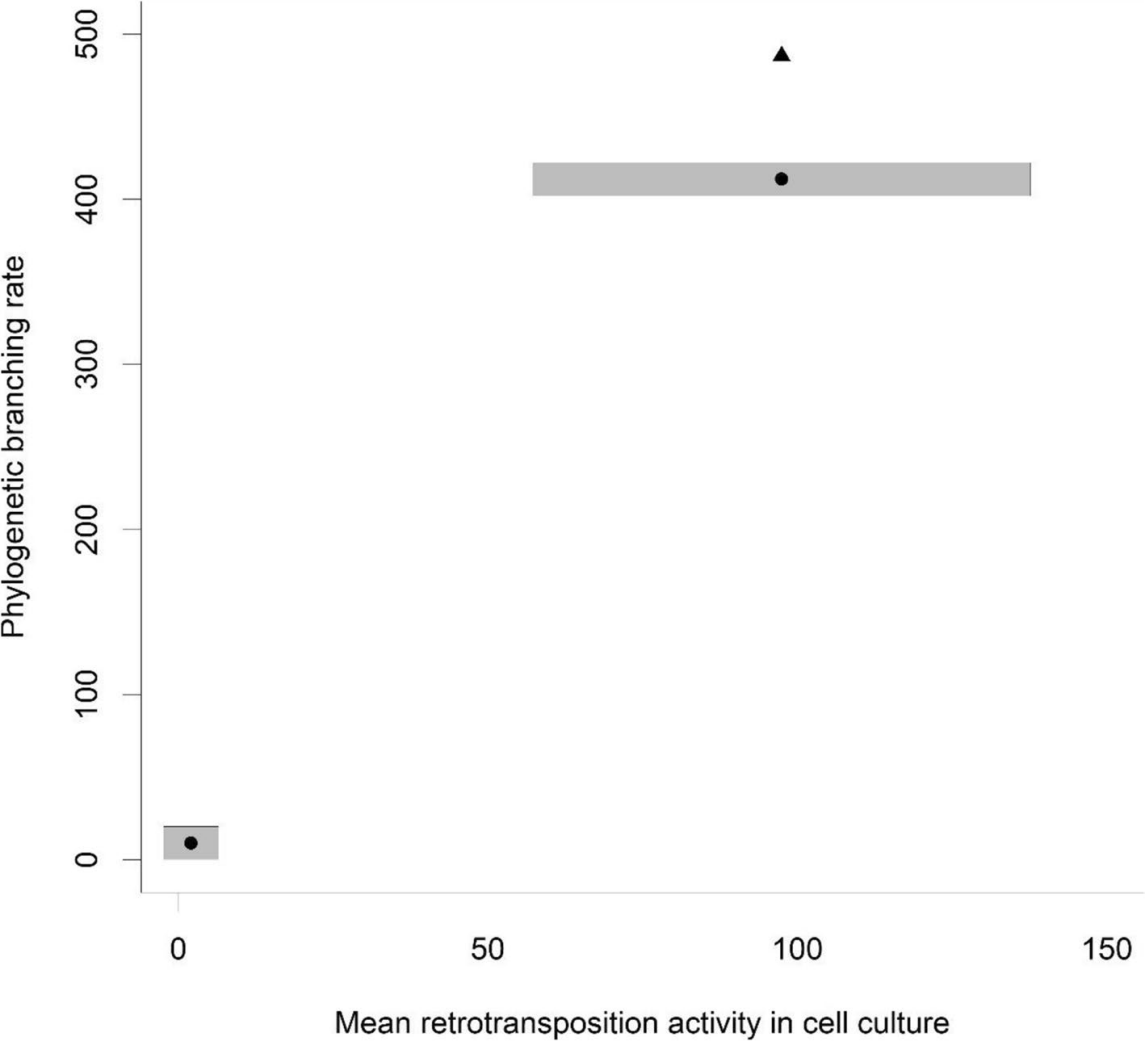
Estimated phylogenetic speciation rates vs. mean retrotransposition activity in cell culture among high and low activity L1s. The rates on the different axes cannot be compared directly since they are measured in different units. The grey bars show the standard deviation of the retotransposition activity (it extends one standard deviation to each side of the point). The values on the y-axis of the circles show the maximum likelihood estimates of the best model in Table 2. The triangle shows what the higher branching rate would have to be for the observed activities and estimated branching rates to have the same ratio between high and low activity L1s

**Fig. 3:**
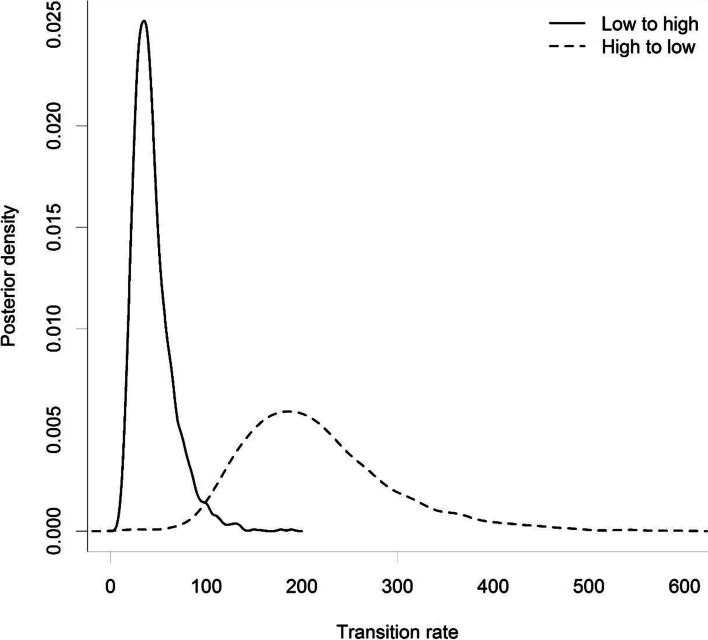
Posterior distribution of the transition rates between high and low retrotransposition activity, obtained from the BayesTraits analysis

The phylogenetic tree of full-length L1s suggests that L1s underwent repeated phylogenetically independent transitions between high and low retrotransposition rates (Fig. 1). The tree also shows that L1s with lower retrotransposition activity values tend to be on longer tips than L1s with higher activity values (Fig. 1). This negative correlation between branch length and retrotransposition rates was confirmed by the results of the BiSSE models. The best-fitting model (i.e. the model with the lowest AIC) implies that high-activity L1s have a speciation rate about 40 times higher than low-activity L1, that L1s generally remain after detection (μ_0_ = μ_1_ = 0) and that *q*_10_, the rate at which high-activity L1s become low-activity L1s is ten times higher than *q*_01_, the rate at which low-activity L1s become a high-activity L1s (Table 2). Nevertheless, the transition rate from low to high activity (*q*_01_) is non-zero, and any model that constrains this transition to zero fits the data substantially worse (Table 2). The ratio of the two estimated speciation rates (λ_0_ / λ_1_) is close to the ratio of mean retrotransposition values from cell cultures among low and high-activity L1s (Fig. 2). According to the parameters of the best-fitting model, the L1 density in the human genome increases at a rate of 127 insertions per nucleotide substitution and an equilibrium proportion of high-activity L1 25%. The proportion of high activity L1 among the sequences analyzed in this dataset is 24.5%. Using a substitution rate of 2.5*10^− 8^ per nucleotide and generation [19], the L1 growth rate becomes 3.2 *10^− 6^ and the according doubling time of the total number of L1s in the genome is 2.2*10^5^ generations. A Bayesian analysis to fit models for the evolutionary transitions between high and low retrotransposition rates yielded a similar asymmetry of evolutionary transitions between high and low retrotransposition activities (Fig. 3). The mean transition rates are 44 for the transition from low to high retrotransposition and 215 for the reverse. The posterior probabilities for equal transition rates between both retrotransposition activities is less than 0.003. The posterior probability for a model that does not allow a transition from low to high retrotransposition is less than 0.0002. The version of the best model with one change-point achieved a slightly higher fit than the constant time model (ΔAIC = 0.43) but the second change point did not improve the fit. According to the one-change point model, both speciation rates (λ_0_ and λ_1_) were 1.6 times higher in the time predating the change point than in the time after the change point. All other aspects of the one-change point model are qualitatively the same as in the best-fitting constant time model. After the change point, the estimated L1 growth rate is 1.4 *10^− 6^ with an L1s doubling time of 5*10^5^ generations.

## Discussion

This study shows that retrotransposition activity values obtained from cell-culture based assays are roughly proportional to the estimated rates at which new L1s emerge in the human population. L1s with higher retrotransposition activities branch more frequently on the phylogenetic tree of human L1s. There appears to be an asymmetry in the evolution of retrotransposition activity, where L1s change more readily from high to low retrotransposition activity than from low to high. This asymmetry was shown by two different analysis methods. These two methods also showed that while low-activity L1s rarely turn into high-activity L1s, the rate for this transition is not zero. In addition, there was evidence for a recent decrease in the rate at which L1s emerge in the human population. When combined, the estimated insertion rates and rates of L1 retrotransposition activity evolution suggest that L1s continue to grow in the human population, albeit at a rate that decreased recently.

Comparative methods, such as the ones used in this analysis, have several limitations. They can provide misleading results when applied to un-replicated evolutionary events [21]. The lack of replication should not be a major concern in our dataset since the L1 tree indicates that there were several phylogenetically independent transitions between high and low retrotransposition activity (Fig. 1). A specific caveat of the BiSSE model is that unaccounted variation in the speciation rate can lead to a spurious correlation between specific character states and the speciation rate [22]. However, this is mainly a problem for analyzing speciation rates of complex organisms where myriads of traits could potentially affect the speciation rate. The context of our analysis is different. For one, L1s are not organisms and therefore harbor fewer traits that could be associated with speciation. Furthermore, cell-culture based retrotransposition estimates directly quantify insertion events. The most parsimonious expectation should therefore be that the speciation rate observed on the L1 phylogenetic tree is proportional to cell-culture based estimates. Our BiSSE results indicate that this expectation is consistent with the data.

There is an additional caveat for applying the BiSSE model to L1 retrotransposition. The BiSSE model interprets each internal node of the phylogenetic tree as a speciation (or in our case retrotransposition) event. The 155 different L1 loci studied in our analysis require 154 retrotransposition events. However, these 154 retrotransposition events do not have to exactly coincide with the 154 internal nodes of the L1 tree, because strictly speaking, the internal nodes correspond to coalescent rather than retrotransposition events and the coalescent process within the human population might be on a comparable time scale as the time between different retrotransposition events. More accurate parameter estimation might therefore require a model that considers the coalescent and retrotransposition process simultaneously. Nevertheless, the ratio of speciation rates estimated via the BiSSE model for high and low retrotransposition L1 is very close to the ratio of retrotransposition rates for these L1 classes obtained from cell cultures, suggesting that the results obtained by ignoring the coalescent process might still be reasonably accurate.

The results of the BiSSE and BayesTraits models provide also information on how retrotransposition activity evolves after insertion. Both approaches show clear statistical support for a model in which the evolutionary change from high to low retrotransposition activity is much more likely than for the reverse. This is consistent with a priori expectations since random mutations of L1 sequences are more likely to disrupt the retrotransposition machinery than improve it. Both approaches indicate that, nevertheless, L1s occasionally change from low activity to high activity. Each model has its own strength and weakness. The BiSSE model requires ultrametric trees, and hence a more restrictive phylogenetic estimation procedure, but it allows incorporating the effects of activity on branching. The BayesTraits model poses no restrictions on the tree branch lengths but does not incorporate the effects of activity on branching. The fact that both models arrive at qualitatively similar conclusions about the evolution of retrotransposition activity underscores the robustness of these results.

Interpretation of the BiSSE parameters requires a careful consideration of the data. Both studies whose data were used in this analysis [9, 10] searched for full-length L1s in a limited set of sample sequences. The first study performed a BLAST search of a full-length L1 sequence against human genomic databases available in 2003 [9]. 44% of the 90 L1s analyzed in this study are polymorphic with an average allele frequency of 44%. The second study searched for non-reference L1s in fosmid clones constructed from genomic DNA of six individuals and only analyzed L1s that occur in at least two fosmid clones [10]. 100% of the 69 L1s identified in the second study are polymorphic with an average allele frequency of 16% [10]. The average allele frequency of all L1s from both studies combined is 62%. By comparison, the average allele frequency of polymorphic full-length L1s in the 1000 genome data is 3% [23]. (Data were obtained from ftp://ftp.1000genomes.ebi.ac.uk/vol1/ftp/release/20130502/, L1s were identified using the tag ““INS:ME:LINE1“ and the tag “SVLEN” was used to select L1s over 6000 bp length). Hence, the majority of L1s included in this analysis are polymorphic and occur at a higher population frequency than the average L1, suggesting that methods to detect L1 in the two studies were biased against low-frequency L1s.

The speciation rates of the BiSSE model estimates the rate at which new L1 insertions occur and reach high enough population frequencies to be detected. As such, the speciation rate in the BiSSE model combines the effects of mutation, selection and drift. Similarly, the rate at which an L1 generates new L1 insertions depends on its retrotransposition activity and population frequency. The estimated transition rates between high and low retrotransposition activity are therefore the result of the combination of two processes, the evolution of retrotransposition activity and changes in allele frequency. On average, full-length L1s have a negative selection coefficient [24] and most likely individual L1 insertions vary widely in their selective effect. The speciation rates estimated here subsume this variation into population-level averages. While these averages ignore a lot of biological complexity, they are sufficient for analyzing population-level dynamics. Since full-length L1s have a negative selection coefficient [24], they depend on drift to increase in population frequency. The smaller the effective population size the more important the relative contribution of drift, and therefore the more likely L1s are to emerge. The human effective population size has changed over time with a bottleneck about 4700 generations ago and population expansion in the last 140 generations [18]. It is therefore likely that the rate at which new L1s reach higher population frequency was higher during the bottleneck and slowed down recently. The change point model confirms a recent decline in the apparent retrotransposition rate that is most likely due to the recent increase of the effective population size.

Since the majority of L1s included in this analysis are polymorphic, the estimated speciation rates are likely to be higher than the allele substitution rate, i.e. the rate at which new alleles arise and become fixed in the population, and lower than the de-novo insertion rate. Estimating the substitution rate would require restricting the model to fixed L1s. However, restricting the analysis to L1s that are fixed in the population would miss the high-activity alleles that tend to be polymorphic and contribute significantly to the overall retrotransposition [9].

The best-fitting BiSSE model restricts the extinction rates to zero (μ_0_ = μ_1_ = 0). There are two possible explanation for these zero extinction rates. Non-zero extinction rates lead to an uptick of the apparent speciation rate in the very recent past, since these are branches that have not yet gone extinct [25]. The zero extinction rate could therefore be an artefact of a decline in drift due to recent population expansion that masked an uptick in apparent speciation rate. Alternatively, the zero extinction could be because low frequency L1s have a low probability to be included in the two studies whose data were used in this analysis. L1s that reached a sufficient population frequency to be detected, might get lost from the population at a rate that is low, relative to the other rates in the BiSSE model. Either way, an extinction rate of zero in the fitted BiSSE model does not contradict a frequent loss of L1s shortly after insertion, because most of these low frequency L1s would not be detected in the studies analyzed here.

According to the BiSSE model, an average full-length L1 generates 3.2 *10^− 6^ new L1 insertions per generation. The model furthermore estimates that at a steady state, 75% of L1s are low activity, leading to an average retrotransposition activity of 27%. Ewing & Kazazian estimated the L1 retrotransposition in humans to be between 1/95 and 1/270 births [26]. Our population-level estimates of insertion rates would be equivalent to the insertion rate per individual if L1 insertions were selectively neutral [27]. In that case, each individual would have to carry on the order of 10^3^ average retrotransposition competent full-length L1s for our estimate to be compatible with the estimate by Ewing & Kazazian. However, the published estimates of the number of L1s with intact ORFs in a human genome range from 90 to 266 [28, 29]. There are several possible reasons for this mismatch in number of active L1s. For one, the ratio of high and low retrotransposition L1s might not yet be in steady state. Furthermore, full-length L1s are under negative selection [24]. Negative selection weeds out many L1s shortly after insertion, which could explain why the insertion rate on the individual level is much higher than a population-level substitution rate. This effect can be even more pronounced when there is a variation in selective effects, so that a certain proportion of L1s are selected out immediately after insertion.

It is unknown whether L1s are growing in the human population or are at a stable equilibrium. Linear models, such as the BiSSE model, only allow for exponential growth or decline. According to our parameter estimates, L1s grow currently exponentially with a doubling time in human genomes of 5*10^5^ generations. It is not clear what mechanism would lead to a negative feedback of L1 density on average retrotransposition rate that is required for a stable equilibrium. It has been suggested that a stable equilibrium for retrotransposition is obtained when the number of available genomic positions becomes limiting and L1s repeatedly insert into pre-existing L1s [7, 8]. However, the low density of active L1s in human genomes makes it unlikely that such a feedback is the driving force for an equilibrium. Alternatively, there might be no equilibrium for the number of L1s but instead co-evolutionary cycles where phases of high L1 retrotransposition lead to evolutionary adaptations in the host that suppress retrotransposition, which in turn increases selection for L1s that can escape the host suppression. There is some empirical evidence for such cycles [30]. A more complete understanding of the L1 dynamics in human genomes will require a model that combines the effects of L1 retrotransposition rate on L1 growth, the evolution of this rate and the fitness effects on the host. The results presented here are a first step in that direction by providing parameter estimates for the first two components.

## Conclusion

The diversification rates estimated from L1 phylogenetic trees are largely consistent with cell culture-based retrotransposition estimates, which validates both methods. The evolutionary decline of retrotransposition rates is supported by two different models and consistent with prior expectation. Hence, applying comparative methods to analyze L1 retrotransposition produces robust and coherent results that provide insight into L1 dynamics on a larger scale. According to our results, active L1s are currently increasing in human genomes.

## Data Availability

This analysis used published data. L1 sequences were obtained from the supplemental material of (https://www.pnas.org/content/suppl/2003/03/31/0831042100.DC1/1042Fig5.pdf) and (https://ars.els-cdn.com/content/image/1-s2.0-S009286741000557X-mmc2.pdf). R code and processed data of the analysis presented here are available at https://osf.io/u6xsr/?view_only=2a81432e729a4c8985c0dbfb07bd16dc.
